# Covered stent assisted coil embolization of large Buhler aneurysm in setting of chronic celiac trunk occlusion

**DOI:** 10.1186/s42155-023-00416-4

**Published:** 2024-01-10

**Authors:** Pietro Quaretti, Riccardo Corti, Antonio Mauro D’Agostino, Antonio Bozzani, Lorenzo Paolo Moramarco, Nicola Cionfoli

**Affiliations:** 1 Interventional Radiology Unit, Fondazione IRCCS Policlinico San Matteo, V.Le Golgi 19, Pavia, 27100 Italy; 2 Vascular and Endovascular Surgery Unit, Fondazione IRCCS Policlinico San Matteo, Pavia, Italy

**Keywords:** Arc of Bühler, Aneurysm, Visceral aneurysm, Stent graft, Embolization

## Abstract

**Background:**

The arc of Bühler (AOB) is a residual embryonal anastomosis between the celiac artery (CA) and the superior mesenteric artery (SMA). Although usually asymptomatic, it has clinical relevance when compensatory reverse flow between the SMA and the CA in response to celiac artery obstruction leads to aneurysm formation and bleeding. Endovascular coiling is the mainstay therapy because of the deep AOB retropancreatic location, which hinders open surgery.

**Case presentation:**

We herein report a case of a 2.8-cm AOB saccular aneurysm and LAM compression of celiac trunk in a 47-year-old man during rehabilitation following motorcycle trauma and vertebral surgery. The patient was considered unsuitable for surgery. Neither conventional coiling nor bare-metal stent and balloon-assisted techniques for coiling were suitable because of the wide necked saccular shape of AOB aneurysm interposed between the SMA and the floor of celiac trunk. To exclude the aneurysm from direct SMA inflow and permit safe and efficient coiling to rule out retrograde sac perfusion, a 9-mm polytetrafluoroethylene stent graft (Viabahn; Gore, Phoenix, AZ, USA) was positioned in the mesenteric artery, followed by antegrade periprosthetic high-density packed coiling of the aneurysm. The AOB remained excluded from mesenteric perfusion. The patient’s clinical condition and abdominal contrast-enhanced multislice computed tomographic findings were unremarkable at the 9-year follow-up.

**Conclusion:**

The 9 year long-term efficacy in our case raises the possibility that perigraft coiling following stent-graft deployment in the SMA may represent a valuable technical option for large Bühler aneurysms that are not amenable to stand-alone coiling.

## Background

The arc of Bühler (AOB) is a rare (estimated frequency, 3%) ventral anastomosis between the celiac artery (CA) and the superior mesenteric artery (SMA) or their branches that do not regress in prenatal life. It is in the retropancreatic space, which hosts the roots of the major vessels. Its existence is beneficial and provides an additional collateral route in cases of pancreaticoduodenal arcade or dorsal pancreatic artery failure. Usually, it can be a worrisome and challenging anatomic variant discovered during pancreaticoduodenal surgery, causing intraoperative difficulties, such as anatomy misidentification or unexpected bleeding [5]. Rarely, AOB aneurysms may develop as a consequence of retrograde high flow in patients with occluded or stenotic CA [3]. Direct coiling is the first-line therapy for asymptomatic [2] and ruptured AOB [1, 8], but anatomic features may jeopardize endovascular treatment owing to the risk of coil migration [3]. Herein, we report a case of a large AOB aneurysm treated by deploying a stent graft in the proximal SMA, followed by aneurysmal sac coiling through a coaxial catheter-microcatheter system jailed between the graft and the native SMA wall.

## Case presentation

**Fig. 1:**
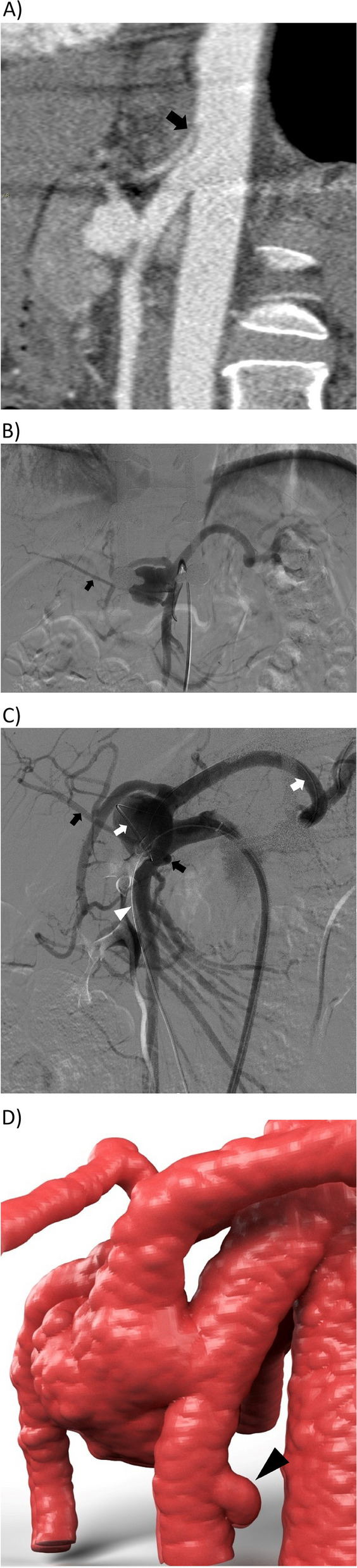
Preoperative imaging of the arch of Bühler (AOB) aneurysm. **A** Contrast sagittal CT-scan showing a 2.8 cm saccular aneurysm (arrowhead) without mural thrombus originating from SMA. Substantial pre-occlusive stenosis of celiac trunk (arrow) by LAM. **B** Pre stenting selective angiography of SMA, in AP projection (accessory right hepatic artery (black arrow)), and oblique projection **C** allows insight into this anatomy variant. A 0.035 “Amplatz guidewire as safety wire is stabilized in SMA (white arrowhead) while the working 0.014”micro-guidewire end in splenic artery (white arrow) after looping inside the aneurysm. A small accessory right hepatic artery (black arrow) is also present. **D** Its origin (arrowhead) on VR3D reconstruction was thereafter covered by Viabahn without consequences

**Fig. 2:**
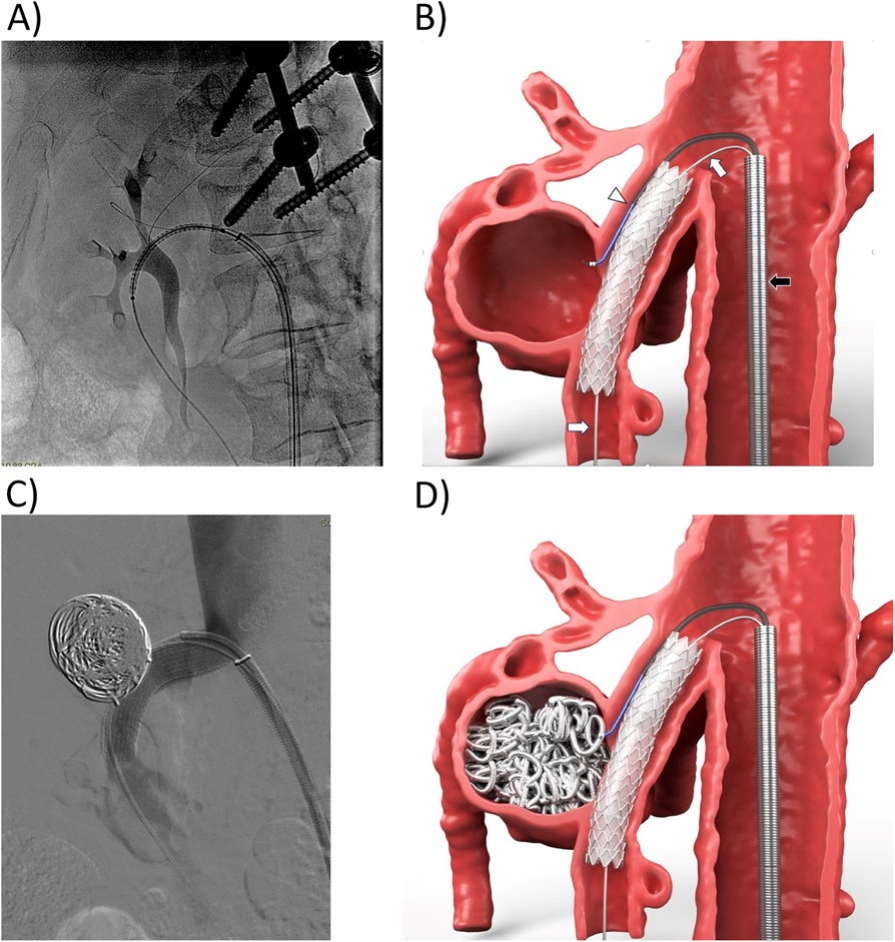
Operative imaging. **A** Angiography in LL projection: Viabahn is positioned over the safety wire before deployment bridging the micro guidewire left inside the aneurysm. Rods and screws for osteosynthesis in vertebral bodies are visible. **B** Schematic drawing after Viabahn deployment. The 5 Fr catheter-microcatheter coaxial system (white arrowhead) is wedged between the stentgraft and the artery wall while the armed 10 Fr sheath ( black arrow) is maintained in place by the safety wire (white arrow) in SMA. **C** Final control angiography. Injection through the sheath after high density packed coiling of the AOB aneurysm on image. **D** Final schematic drawing of aneurysm exclusion

**Fig. 3:**
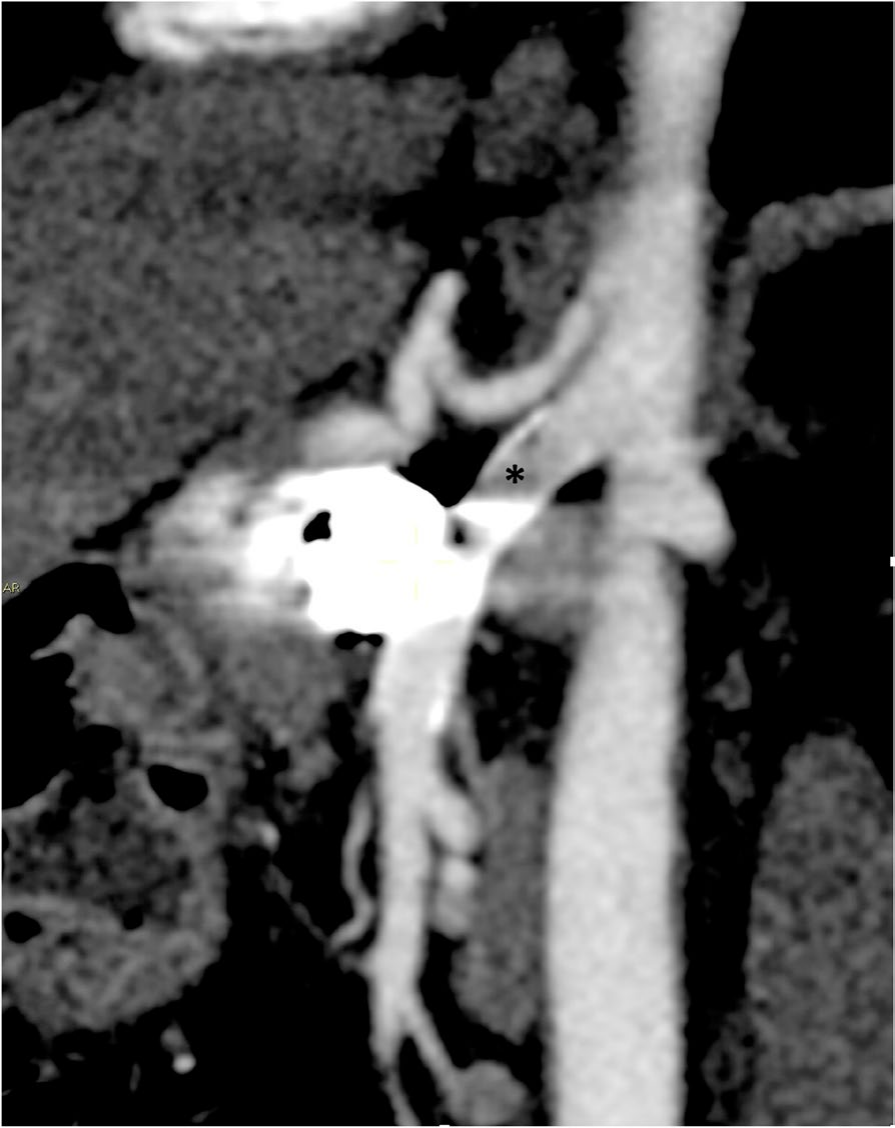
Contrast CT-scan sagittal reconstruction of SMA at 9 year follow-up showing the Viabahn patency. Imaging is noised by coils and orthopedic devices (*). Patient remains asymptomatic

A 47-year-old man was referred to our interventional radiology department for an incidentally discovered visceral aneurysm. Five months prior, he had reported somatic fractures of T12 and L1 as a consequence of a motorbike accident and was treated with vertebral fusion with rods and screws. Contrast multislice computed tomography performed during rehabilitation revealed a 2.8-cm saccular aneurysm directly connecting the proximal SMA with the CA, which was critically compressed by the median arcuate ligament (Fig. 1). The patient was hemodynamically stable (BP, 128/86; HR, 77) and asymptomatic, and laboratory test results were within normal limits. The patient was diagnosed with Bühler aneurysm. The endovascular approach was chosen after a multidisciplinary consultation with abdominal and vascular surgeons and patient counseling. Right transfemoral aortography was performed under local anesthesia. After a failed attempt to catheterize the CA, an injection after SMA engagement showed a saccular AOB aneurysm interposed between the superior wall of the SMA and the inferior floor of the post-stenotic CA. A retrograde high flow running from the SMA to the common hepatic and splenic arteries was observed. The aneurysm involved a 3-cm-long AOB. No takeoff or landing zone for direct stent-graft sealing of the Bühler aneurysm was observed in this anatomic presentation. Conventional coiling was attempted; however, the detachable coils prolapsed from the aneurysm and were withdrawn before detachment. At this point, the sheath was upsized to a 45-cm-long 10-Fr armed sheath (Super Arrow-Flex, Arrow) (Fig. 2). A self-expandable 9 × 50-mm polytetrafluoroethylene (PTFE) stent graft (Viabahn; Gore, Phoenix, AZ, USA) was deployed in the SMA in the LL projection. The length of the Viabahn was chosen to exclude the AOB while preserving the flow in the pancreaticoduodenal arcades. A 0.035-inch Amplatz guidewire was left as a safety wire in the SMA through the SG. Then, a pre-curved 5-Fr catheter (Glidecath; Terumo) was advanced parallel to the safety wire and wedged between the Viabahn and the native wall. A 2.7-Fr microcatheter (Progreat; Terumo, Ja) pre-charged with a 0.014-inch Pilot guidewire was then coaxially advanced through the 5-Fr catheter alongside the stent graft in a periprosthetic manner. Once the Progreat tip was inside the saccular aneurysm, framing and packed coiling of the AOB aneurysm were achieved by releasing an overall length of 385 cm of detachable coils (Ruby coil with three-dimensional shape and soft configuration; Penumbra, Alameda, CA, USA). A 9 × 20-mm angioplasty balloon was then inflated at a low pressure inside the Viabahn for better wall apposition. Completion angiography showed a patent mesenteric stent graft with retrograde opacification of the CA and no residual backflow inside the aneurysm. Femoral hemostasis was achieved using an 8-Fr Angio-Seal (Terumo). The patient was discharged 2 days later, and he was given dual antiplatelets for 3 months and lifelong cardioaspirin. Follow-up clinical and instrumental imaging tests were scheduled, and the last computed tomography angiogram performed 9 years later ruled out aneurysm enlargement or reperfusion with regular patency of the Viabahn and CAs (Fig. 3).

## Discussion

We treated successfully a large saccular AOB aneurysm with a stengraft-assisted coiling.

The AOB can cause bleeding and death [1]. Open surgery has been reported; however, the retropancreatic position of the AOB makes surgical packing or visceral bypass creation challenging [3, 6]. Endovascular therapy is the first-line treatment for pathological AOB. Its usual configuration as a mesenteric collateral of variable length favors embolization therapy [2]. Both conventional coiling for small aneurysms [1] and vessel sacrifice [8] have been used. In the present case, the saccular aneurysm, similar to the AOB treated by Kugai et al. after two failed embolizations with open surgery [3], was not amenable to unassisted coiling owing to the wide neck and forceful flow through the aneurysm. Balloon remodeling was the first technique considered. This option was excluded because of the risks of dissection, thrombosis, and embolism in the parent artery inherent in prolonged ballooning in the SMA. Bare-metal stent (BMS)–assisted coiling seemed to be more appealing in this regard, but it would not have excluded at all the high intra-aneurysmal flow during coiling, with related risks of distal coil migration, being absent a close dome in this aneurysm conformation. Further perceived concerns were the early risk of coil herniation through the interstices of not dedicate open mesh self-expandable stents required by the large diameter of SMA and the late risk of in-stent BMS restenosis and mesenteric ischemia. Ultimately, we opted to deploy a self-expandable heparin-bonded PTFE stent graft (Viabahn; Gore) to exclude any residual mesenteric direct flow within the AOB, facilitating safer and more effective coil packing. Furthermore, because of the likely better patency of SG than that of BMS in the digestive arteries, the risk of patency loss appeared to be almost halved using a covered stent [7]. Considerable attention was given to the SMA calibration to ensure proper oversize when choosing the diameter of the SG, regardless of bore access. A length of 5 cm was allowed to bridge the takeoff of the AOB while keeping the pancreatic duodenal arcade patent for collateral flow. Nevertheless, coiling was deemed necessary to impede the retrograde pressurization of the AOB. Intraoperative or postoperative periprosthetic EVAR sac embolization by coiling has been proposed to prevent or cure type II endoleaks, with promising results in high-risk situations for type II leaks [4]. To the best of our knowledge, periprosthetic coiling for visceral aneurysms has not been proposed yet. In the present case, it seemed to be a valuable technical option considering the anatomic features of the AOB and the physical properties of the Viabahn. The periprosthetic engagement and navigation of the curved catheter were performed in a straightforward manner. Stent-graft–assisted high-density coiling was performed. In the case of failed antegrade periprosthetic navigation, the AOB aneurysm could have been retrogradely coiled through the longer duodenal arcades.

## Conclusion

We herein report perigraft coiling of a large AOB aneurysm following stent-graft deployment in the SMA. Safety concerns about the deployment of stent grafts in visceral vessels require that this endovascular technique be used only for large AOB aneurysms not amenable to stand-alone coiling.

## Data Availability

Not applicable.

## Abbreviations

AOB: Arc of Bühler
BMS: Bare-metal stent
CA: Celiac artery
PTFE: Polytetrafluoroethylene
SG: Stent graft
SMA: Superior mesenteric artery
